# Robotic locomotor training for spasticity, pain, and quality of life in individuals with chronic SCI: A pilot randomized controlled trial

**DOI:** 10.3389/fresc.2023.1003360

**Published:** 2023-01-30

**Authors:** Claire Shackleton, Robert Evans, Sacha West, Wayne Derman, Yumna Albertus

**Affiliations:** ^1^ Department of Human Biology, Physical Activity, Lifestyle and Sport Research Centre (HPALS), University of Cape Town, Cape Town, South Africa; ^2^ Department of Sport Management, Cape Peninsula University of Technology, Cape Town, Western cape, South Africa; ^3^ Institute of Sport and Exercise Medicine, Faculty of Medicine and Health Sciences, Stellenbosch University, Tygerberg Campus, Cape Town, Western cape, South Africa; ^4^ International Olympic Committee Research Center, IOC Research Center, Cape Town, South Africa

**Keywords:** spinal cord injuries, exoskeleton device, exercise therapy, pain, muscle spasticity, quality of life

## Abstract

**Objective:**

The prevention and treatment of secondary complications is a key priority for people with spinal cord injury and a fundamental goal of rehabilitation. Activity-based Training (ABT) and Robotic Locomotor Training (RLT) demonstrate promising results for reducing secondary complications associated with SCI. However, there is a need for increased evidence through randomized controlled trials. Therefore, we aimed to investigate the effect of RLT and ABT interventions on pain, spasticity, and quality of life in individuals with spinal cord injuries.

**Methods:**

Participants with chronic motor incomplete tetraplegia (*n* = 16) were recruited. Each intervention involved 60-minute sessions, 3× per week, over 24-weeks. RLT involved walking in an Ekso GT exoskeleton. ABT involved a combination of resistance, cardiovascular and weight-bearing exercise. Outcomes of interest included the Modified Ashworth Scale, the International SCI Pain Basic Data Set Version 2, and the International SCI Quality of Life Basic Data Set.

**Results:**

Neither intervention altered symptoms of spasticity. Pain intensity increased from pre-post intervention for both groups, with a mean increase of 1.55 [−0.82, 3.92] (*p* = 0.03) and 1.56 [−0.43, 3.55] (*p* = 0.02) points for the RLT and ABT group, respectively. The ABT group had an increase in pain interference scores of 100%, 50%, and 109% for the daily activity, mood, and sleep domain, respectively. The RLT group had an increase in pain interference scores of 86% and 69% for the daily activity and mood domain respectively, but no change in the sleep domain. The RLT group had increased perceptions of quality of life with changes of 2.37 [0.32, 4.41], 2.00 [0.43, 3.56] and 0.25 [−1.63, 2.13] points, *p* = 0.03, for the general, physical, and psychological domains, respectively. The ABT group had increased perceptions of general, physical and psychological quality of life with changes of 0.75 [−1.38, 2.88], 0.62 [−1.83, 3.07] and 0.63 [−1.87, 3.13] points, respectively.

**Conclusions:**

Despite increased pain ratings and no change in symptoms of spasticity, there was an increase in perceived quality of life for both groups over 24-weeks. This dichotomy warrants additional investigation in future large-scale randomized controlled trials.

## 1. Introduction

The complex and widespread physiological consequences of a spinal cord injury (SCI) and the associated physical inactivity can lead to increased risk of secondary health complications (1–3). Both spasticity and chronic pain are common secondary complications, affecting approximately 80% of individuals with SCI (1, 4–8). Spasticity and pain are rated as some of the most debilitating secondary consequences of SCI, causing significant physical disability, restricting independence and activities of daily living (ADLs), and reducing quality of life (QoL) (1, 4, 8–10). Additionally, these secondary complications are a frequent cause of morbidity and mortality for people with SCI and can lead to increased rates of rehospitalization, increased medical costs, loss of employability and social engagement with resultant decreased psychological well-being (1, 2, 11).

Therefore, the prevention and treatment of secondary complications is a key priority for people with SCI and a fundamental goal of SCI rehabilitation (1, 12). Regular engagement in physical activity can attenuate the risk of developing secondary complications in the SCI population (8, 10). Activity-based Training (ABT) utilises regular standing, aerobic, and resistance exercises to aid in the prevention and management of secondary complications (13–15). There is preliminary evidence that an alternative exercise therapy, Robotic Locomotor Training (RLT), also has beneficial effects on spasticity and pain in this population (16, 17). Although these results are promising, continued experimental research is required to study the extent to which RLT mitigates comorbidity risk, so that exercise guidelines can be developed to prevent these conditions and improve overall QoL (3). Thus, this study aimed to determine the effects of RLT compared to conventional ABT on spasticity, pain and QoL experienced by individuals with SCI during a pilot randomized controlled trial.

## 2. Materials and methods

### 2.1. Participants

**Figure 1:**
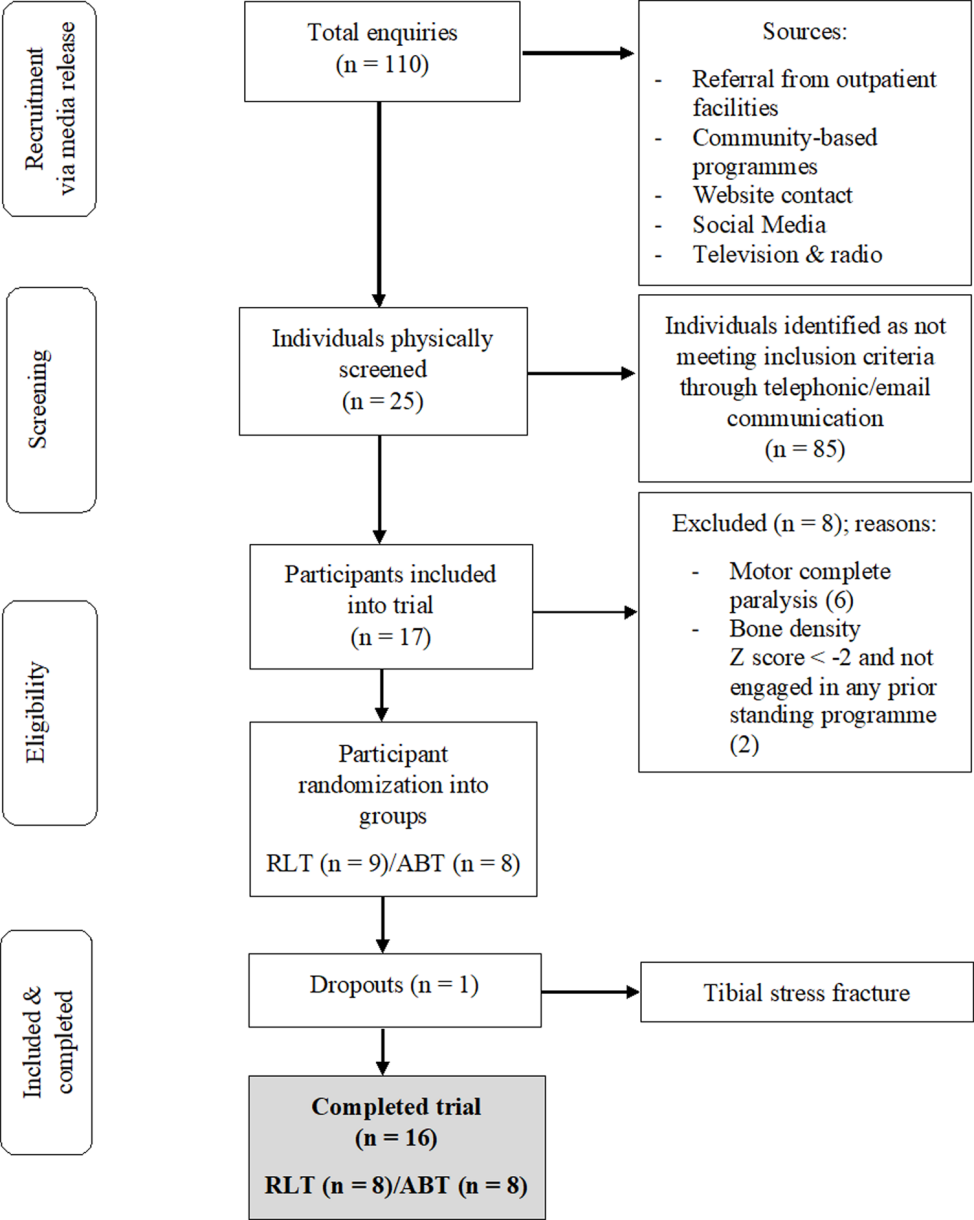
CONSORT flow chart of the recruitment process of participants into the trial.

This study is a secondary analysis from a pilot RCT, for which information on recruitment, adherence, methods and sample size determination have previously been reported and published (18, 19). A total of 17 participants with chronic [>1 year] traumatic, motor incomplete tetraplegia were recruited and assigned *via* random number generation to the RLT or ABT intervention groups (Figure 1). Detailed inclusion and exclusion criteria are contained in Supplementary Table S1. Each participant provided written informed consent prior to the study. The study was approved by the XX Human Research Ethics Committee (Ref: XX) and has been registered on the XX Clinical Trials Registry (XX). A post-trial care period of three months was implemented after participants finished the intervention, with continued access to rehabilitation equipment and medical professionals provided.

### 2.2. Rehabilitation interventions

An overview of the testing timeline, procedures and intervention has been previously described (19). Both interventions consisted of three sessions per week, 60-minutes each, for 24-weeks and were overseen by trained exercise therapists. RLT involved solely walking in an Ekso® GT exoskeleton [Ekso Bionics, Richmond, CA, US]. Intensity levels were determined by the attending therapist and ranged from standing and walking time of 10 to 50 min and between 50 and 1,800 steps taken.

The ABT intervention was adapted from the *Beyond Therapy* model used by the Shepherd Centre (15). ABT consisted of a combination of resistance, cardiovascular, and flexibility training in various positions as well as gait retraining, without a treadmill or robotic assistance. Upper and lower body resistance training was performed using bodyweight exercises and various apparatus, including bands, wrist weights, dumbbells, and cables. The approximate standardised time allocation for each ABT session was as follows: warm-up and mobility [5 min], resistance training [20–30 min], and cardiovascular training [20–30 min]. Five minutes were allocated for transfers and the setting up of various apparatus. Participants' physical activity levels were monitored using the PARA-SCI (20) tool and were advised not to change their physical activity habits outside of the trial.

### 2.3. Testing procedures

All 16 participants underwent evaluations of spasticity and completed the pain and QoL questionnaires at baseline, 6, 12, and 24 weeks. This study is a secondary analysis of a larger pilot trial that assessed functional capacity and cardiovascular outcomes (18, 19). Specific methods pertaining to the secondary complication assessments are provided below:

#### 2.3.1. Spasticity evaluation: Modified Ashworth Scale

Spasticity was measured in all participants using the Modified Ashworth Scale (MAS) developed by Bohannon and Smith in 1986 (21). The MAS has been shown to be a reliable tool in SCI populations (21) and it is the most widely used assessment tool to measure resistance to limb movement in a clinical setting (22). Assessment techniques were standardized, including the test positions, right–left test order, speed of assessment and adequate training within a single rater.

#### 2.3.2. Pain questionnaire: International SCI pain basic data set version 2

Due to the subjective nature of pain, an individual's perception of his/her pain is essential for a comprehensive evaluation of pain after SCI (23). The validated International SCI Pain Basic Data Set Version 2 (24) was used to determine the intensity and location of pain that participants experienced, and the subsequent impact of that pain interference on three domains: (A) Daily activities; (B) Mood; (C) Sleep.

#### 2.3.3. Quality of life questionnaire: International SCI quality of life basic data set

Self-report measures are widely used in the SCI literature to assess a participant's mental and emotional state (25). The validated International SCI Quality of Life Basic Data Set (26) was used to assess average QoL over the last month across three domains: (A) life as a whole (general life); (B) physical health; (C) psychological well-being. Scores were rated on a scale ranging from 0 (completely dissatisfied) to 10 (completely satisfied). A higher score indicates greater perceptions of QoL.

### 2.4. Statistical analysis

All data were analysed using statistical software (R, R Core Team, Auckland, New Zealand and Prism 8, GraphPad Software Inc, California, USA). Normality was assessed using histograms and plots to validate the models. Linear mixed effect models assessed continuous responses which were measured at four time points [0, 6, 12 and 24-weeks]. These mixed effects models formally compared the effect of the group (ABT vs. RLT) interventions across the total 24-week period and tested for any changes over time (pre vs. post). Due to small sample size, it was not possible to fit nonlinear time trends; hence only a linear time effect over the entire 24-week period was considered. To account for the within-subject association between repeated measures, subject specific random effects were included (modelled coefficient *p*-values and 95% CIs). Response profiles were illustrated using plots of means and half-width 95% confidence intervals (CI) for observed data. Significance was accepted at a *p* < 0.05. Magnitude-based inferences of change (effect size) were calculated according to Cohen's d (27) to show estimates for observed significant differences. A Cohen's d of zero denotes no effect, whereas ranges from 0.2–0.5, 0.5–0.8 and >0.8 represent small, medium and large effects, respectively (27).

## 3. Results

### 3.1. Participant characteristics

A total of 16 participants, aged 19–60 (mean ± SD: 38.4 ± 14.3 years), completed the trial (Table 1). The RLT and ABT groups were matched at baseline for age and time since injury. Motor vehicle accidents accounted for 63% of injury aetiology, whilst stabbing, gunshot, rugby, motorcycle, mountain bicycle and diving accounted for 12.5% each. One participant discontinued the intervention after being enrolled in the RLT group for three weeks. Persistent right leg weakness necessitated a magnetic resonance imaging study (MRI) which provided images consistent with the diagnosis of a tibial stress fracture. Only baseline measures had been recorded for the participant which may have been confounded by an existing stress fracture. Thus, the participant was excluded from all analyses and received treatment for the fracture outside of the trial protocol. No other adverse events or negative side effects were experienced.

**Table 1:** Participant characteristics of the Robotic Locomotor Training and Activity-based Training groups.

Group	Participant	Age (years)	Time since injury (years)	Neurological level of injury	AIS category	Aetiology	Sex
RLT	1	27	9	C6	D	Stabbing	Male
	2	33	15	C6	C	MVA	Male
	3	32	3	C5	D	MVA	Male
	4	46	26	C4	D	Gunshot	Male
	5	55	4	C5	D	MVA	Male
	6	43	23	C6	C	MVA	Male
	7	56	15	C4	C	MVA	Male
	8	32	15	C7	C	Sport - Rugby	Male
	Average	40.5 ± 11.2	13.8 ± 8.2				
ABT	9	26	2	C6	C	MVA	Male
	10	46	20	C6	D	MVA	Female
	11	50	8	C7	D	MVA	Male
	12	19	2	C5	C	MVA	Male
	13	47	3	C4	D	Motorcycle	Male
	14	29	10	C5	C	MVA	Male
	15	60	2	C5	C	Mountain bike	Male
	16	30	11	C4	C	Diving	Male
	Average	38.4 ± 14.3	7.3 ± 6.4				

*RLT,* Robotic locomotor training (*n* = 8); *ABT,* Activity-based training (*n* = 8); *MVA,* motor vehicle accident. Values quoted as mean ± SD. No statistically significant difference between groups for age (*p* = 0.74) and time since injury (*p* = 0.10).

### 3.2. Spasticity

**Figure 2:**
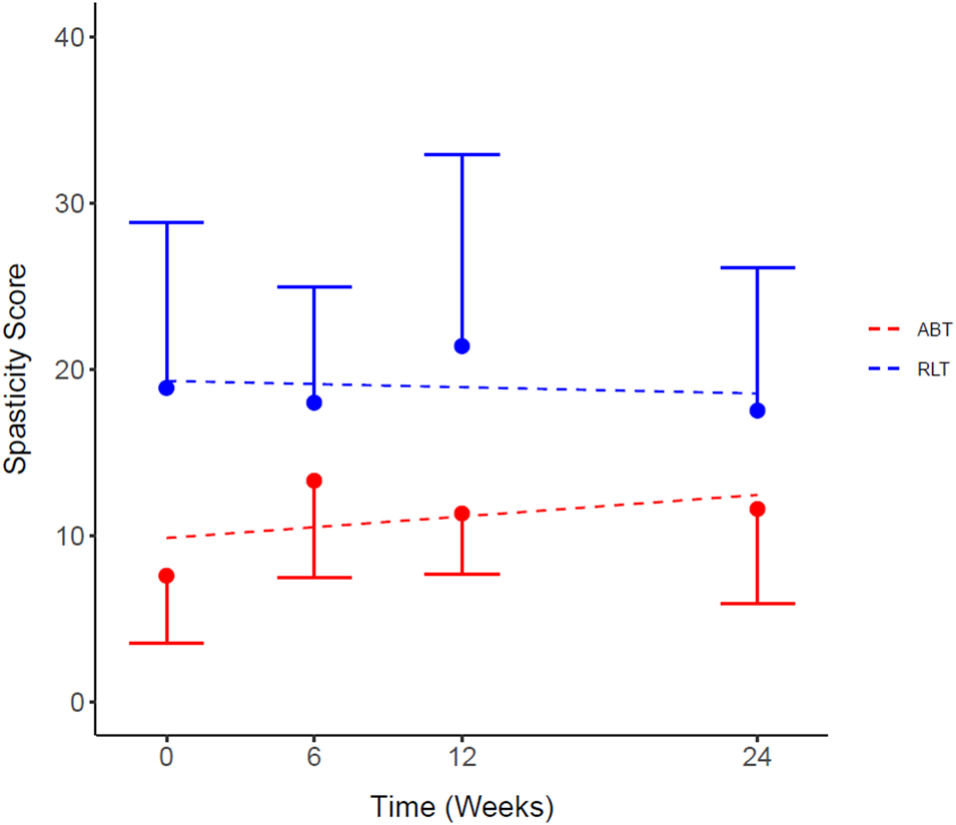
Total spasticity scores for the Robotic Locomotor Training and Activity-based Training groups over time. *RLT*: Robotic Locomotor Training (*n* = 8); *ABT,* Activity-based Training (*n* = 8); *Spasticity score,* sum of scores for 22 tested body areas (combined right and left side) using Modified Ashworth Scale. Data presented as observed mean ± half-width 95% CI. Modelled linear estimates shown as superimposed lines (predicted mean). *No significant differences in spasticity scores at baseline (*p* = 0.09).

Whole body spasticity scores were calculated from the individual scores of the 22 measured body areas. Figure 2 shows that the modelled difference in total spasticity at baseline between the groups was 9.45 units, a non-significant difference (*p* = 0.09). There was no significant difference in total spasticity scores between the RLT and ABT groups over time (*p* = 0.25; ES = 0.57) (Table 2).

**Table 2:** Secondary complications for the Robotic Locomotor Training and Activity-based Training groups at baseline and week 24.

	RLT				ABT				
	Pre	Post	Δ [95% CI]	Δ (%)	Pre	Post	Δ [95% CI]	Δ (%)	Effect size
Spasticity									
Total body	18.88 ± 14.41	17.56 ± 12.29	−1.32 [−13.44, 15.68]	−10	7.62 ± 5.93	11.62 ± 8.25	4.00 [−3.70, 11.70]	52	0.57
Upper body	4.56 ± 6.68	4.44 ± 7.03	−0.12 [−7.23, 7.43]	−3	1.75 ± 2.55	2.12 ± 1.81	0.37 [−2.00, 2.74]	21	0.45
Lower body	14.31 ± 10.95	13.12 ± 8.44	−1.19 [−9.29, 11.67]	−8	5.88 ± 4.16	9.50 ± 7.37	3.62 [−2.79, 10.03]	62	0.46
Pain									
Intensity	4.15 ± 2.71	5.70 ± 1.56	1.55 [−0.82, 3.92]	26	4.02 ± 2.16	5.58 ± 1.50	1.56 [−0.43, 3.55]	46	0.08
Activity interference	2.33 ± 1.80	4.33 ± 1.94	2.00 [−0.01, 4.01]	86	2.38 ± 2.07	4.75 ± 2.92	2.37 [−0.34, 5.08]	100	0.17
Mood interference	2.56 ± 2.79	4.33 ± 2.00	1.77 [−0.83, 4.37]	69	2.25 ± 2.60	3.38 ± 2.72	1.13 [−1.72, 3.98]	50	0.40
Sleep interference	3.11 ± 4.28	3.11 ± 3.02	0.00 [−3.97, 3.97]	0	1.38 ± 1.51	2.88 ± 2.10	1.50 [−0.46, 3.46]	109	0.09
Quality of life									
General QoL	6.25 ± 2.31	8.62 ± 1.40	2.37 [0.32, 4.41]	27	6.75 ± 2.31	7.50 ± 1.60	0.75 [−1.38, 2.88]	10	0.75
Physical QoL	6.62 ± 1.69	8.62 ± 1.19	2.00 [0.43, 3.56]	23	6.50 ± 2.27	7.12 ± 2.30	0.62 [−1.83, 3.07]	9	**0** **.** **82**
Psychological QoL	8.00 ± 1.69	8.25 ± 1.83	0.25 [−1.63, 2.13]	3	7.12 ± 2.53	7.75 ± 2.12	0.63 [−1.87, 3.13]	8	0.26

*RLT,* Robotic locomotor training (*n* = 8); *ABT,* activity-based training (*n* = 8); *Pre,* week 0 measurement; *Post:* week 24 measurement. *Total spasticity score*: sum of scores for 22 tested body areas (combined right and left side) using Modified Ashworth Scale; *Upper body spasticity score:* sum of scores for 4 tested body areas; *Lower body spasticity score:* sum of scores for 7 tested body areas. *Pain intensity and interference:* Ratings of 0–10 on International SCI Pain Basic Data Set Version 2*; Quality of life (QoL):* Ratings of 0–10 on International SCI Quality of Life Basic Data Set for three domains. Data presented as mean ± SD; Δ *(95% CI);* mean difference ± 95% confidence interval*; % Δ:* mean percentage change from pre to post. Effect size: bold represents large effect size.

### 3.3. Pain

**Figure 3:**
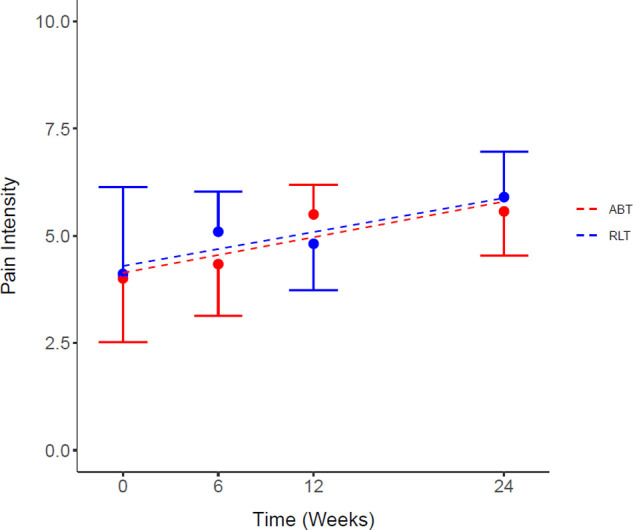
Average pain intensity score for the Robotic Locomotor Training and Activity-based Training groups over time. *RLT,* Robotic Locomotor Training (*n* = 8); *ABT,* Activity-based Training (*n* = 8); *Pain intensity score (0–10),* averaged over number of pain locations reported in the International SCI Pain Basic Data Set Version 2. Data presented as observed mean ± half-width 95% CI. Modelled linear estimates shown as superimposed lines (predicted mean).

The responses to the pain questionnaire demonstrated 81% (*n* = 13) of the participants experienced pain at baseline, whereas by week 24, 100% (*n* = 16) of the participants experienced pain. There was no significant difference between the RLT and ABT groups for pain intensity (*p* = 0.67; ES = 0.08) (Figure 3). However, there was a significant increase in pain intensity from pre to post intervention for both groups, with a mean increase of 1.55 [−0.82, 3.92] (37%) (*p* = 0.03) and 1.56 [−0.43, 3.55] (39%) (*p* = 0.02) for the RLT and ABT group, respectively (Table 2).

**Figure 4:**
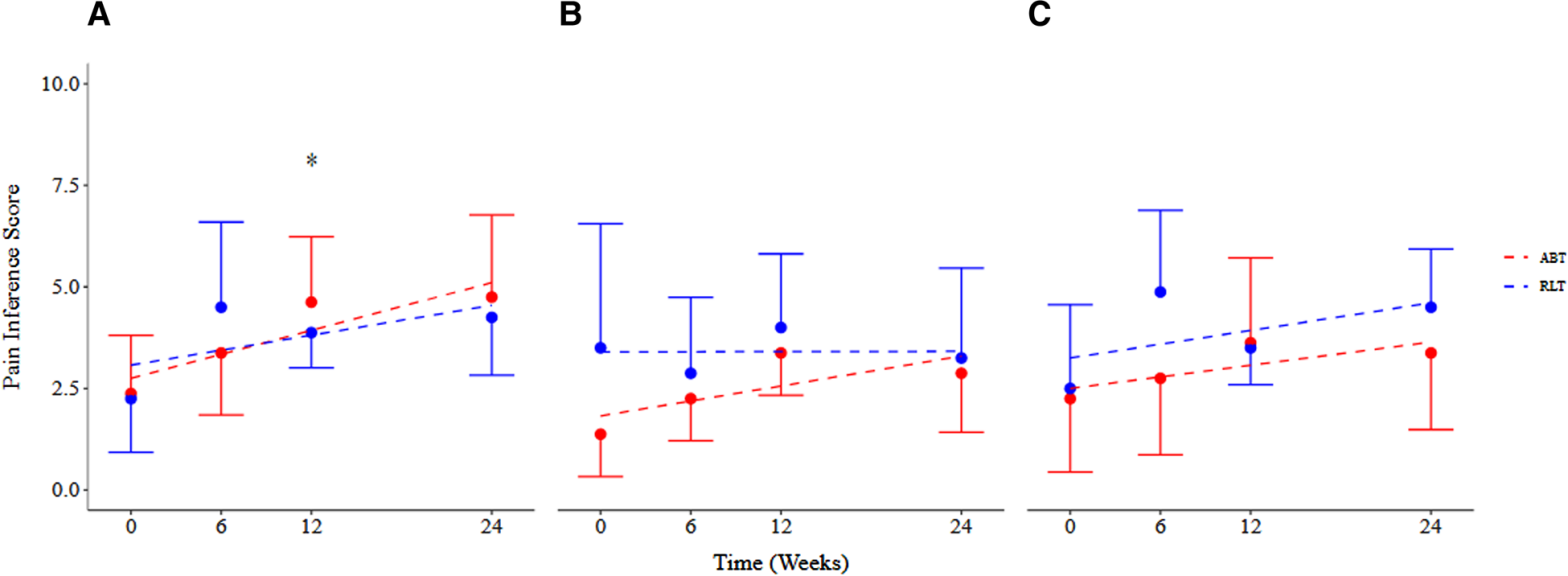
Pain interference scores across (**A**) daily activity, (**B**) sleep, and (**C**) mood domains for the Robotic Locomotor Training and Activity-based Training groups over time. *RLT,* Robotic Locomotor Training (*n* = 8); *ABT,* Activity-based Training (*n* = 8); *Pain interference score (0–10),* International SCI Pain Basic Data Set Version 2; *Pain intensity score (0–10),* averaged over number of pain locations reported in the International SCI Pain Basic Data Set Version 2. Data presented as observed mean and half-width of 95% CI. Modelled linear estimates shown as superimposed lines (predicted mean). *Significant increase in pain experienced in the daily activity domain over time (*p* = 0.05).

There was no significant difference between the RLT and ABT groups over time for pain interference ratings (*p* = 0.61; ES < 0.80). However, both groups reported pain to interfere in an increasing manner with the three measured domains over time (Figures 4A–C). There was a significant time effect for pain interference for the daily activity domain (Figure 3A) (*p* = 0.05) with an increase in pain interference ratings of 2.00 [−0.01, 4.01] and 2.37 [−0.34, 5.08] for the RLT and ABT group, respectively. The ABT group had an increase in pain interference scores of 100%, 50%, and 109% for the daily activity, mood, and sleep domain, respectively. The RLT group had an increase in pain interference scores of 86% and 69% for the daily activity and mood domain respectively, but no change in the sleep domain (0% change) (Table 2).

The highest overall pain was experienced in the shoulder for the ABT group and the lower back for the RLT group (Table 3). The ABT group experienced greater reported pain in various areas, with the neck and shoulder areas dominating. The RLT group experienced noticeable variability in the reported pain areas over time, with only the lower back and upper arm being reported as pain areas at both week 0 and week 24.

**Table 3:** Primary pain complaint area reported for the Robotic Locomotor Training and Activity-based Training groups at baseline and week 24.

	ABT		RLT	
Pain area	Pre	Post	Pre	Post
Shoulder	4	3		1
Neck	1	3	1	
Lower back	1		2	2
Buttocks	1	1	1	
Wrist		1		
Upper arm			2	1
Upper leg/thigh				2
Foot/toe				1
Hip				1
None	1		2	

*RLT*, Robotic Locomotor Training (*n* = 8); *ABT,* activity-based training (*n* = 8); *Pre,* week 0 measurement; *Post,* week 24 measurement; Data presented as frequency count of number of participants that reported that area of pain. *Pain areas:* Listed in the International SCI Pain Basic Data Set Version 2. *Note:* Additional body areas were available for selection on the pain questionnaire, but data above only presents those that were reported.

### 3.4. Quality of life

**Figure 5:**
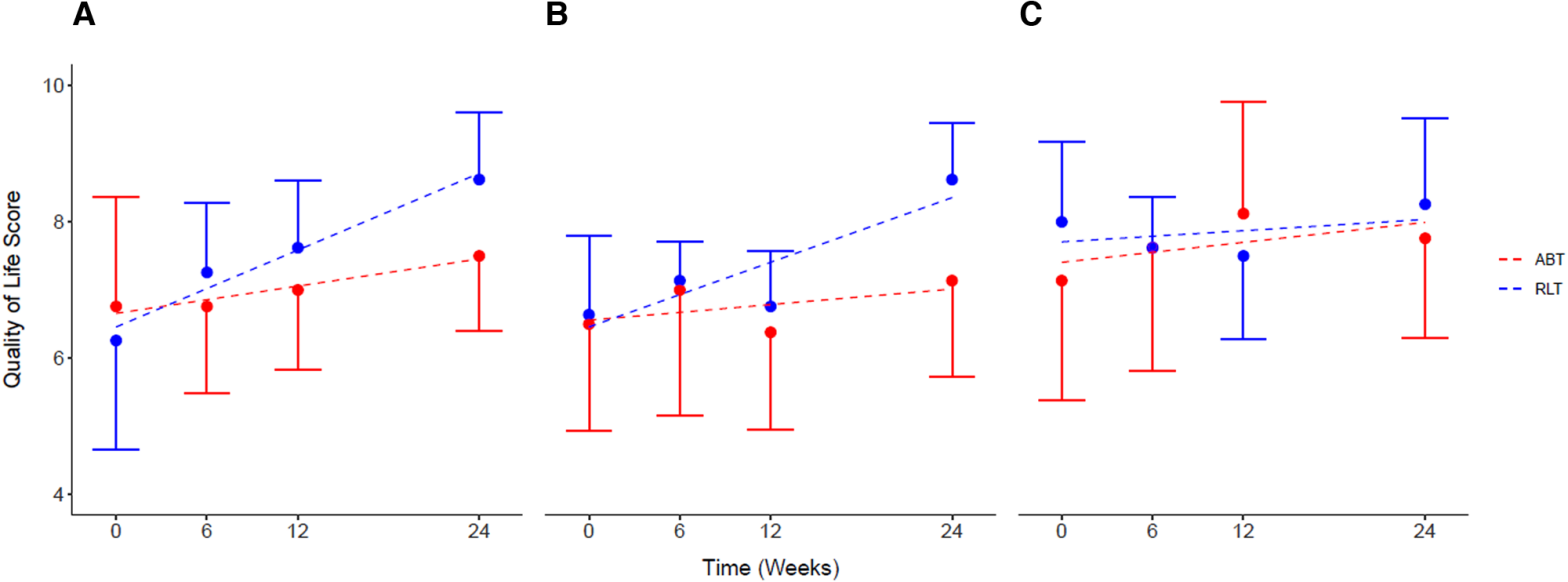
Self-reported quality of life for (**A**) general life, (**B**) physical health, and (**C**) psychological health, for the Robotic Locomotor Training and Activity-based Training groups over time. *RLT,* Robotic Locomotor Training (*n* = 8); *ABT,* Activity-based Training (*n* = 8); *Quality of life score (0–10),* International SCI Quality of life (QOL) Basic Data Set; (**A**): life as a whole; (**B**)*:* physical health; (**C**): psychological health. Data presented as observed mean ± half-width 95% CI. Modelled linear estimates shown as superimposed lines (predicted mean).

There was no significant difference for perceptions of general QoL (*p* = 0.16; ES = 0.75) and psychological QoL (*p* = 0.26; ES = 0.62) between the groups by week-24. However, the large effect size of ES = 0.82 (*p* = 0.12) indicated a group difference in perceptions of physical QoL after 24 weeks of training, with a change of 2.00 [0.43, 3.56] for the RLT group and 0.62 [−1.83, 3.07] for the ABT group post training. Both the ABT and RLT groups showed increasing perceptions of QoL over time, across all three domains: general life, physical health, and psychological well-being (Figure 5). The RLT group had increased (*p* = 0.03) perceptions of general, physical and psychological QoL with changes of 27%, 23% and 3% from pre to post intervention, respectively. The ABT group had non-significant increases of 10%, 9% and 8% for the three domains, respectively (Table 2).

## 4. Discussion

This study aimed to describe the effects of RLT compared to ABT on secondary complications, specifically spasticity, pain and QoL. This is a pilot study that needs to be followed up with a high powered, large-scale RCT.

The first important finding is increased pain ratings for both groups over time. Chronic pain after SCI has been found to be prevalent in approximately four out of five individuals with SCI (1). Thus, it is not surprising that most of the participants in this study experienced pain at baseline and throughout the study. The high prevalence and increasing intensity of pain observed in this study is consistent with previous results documented in a SCI population (23, 28). The increased perceptions of pain within this study could possibly be due to the high intensity and frequency of exercise sessions within the groups. It is well documented that this type of training can cause increased fatigue and chronic muscle or joint pain due to an increase in exercise load over time (29, 30). Another plausible explanation for the increasing perceptions of pain was due to an increase in musculoskeletal pain caused by muscle stiffness above the level of injury (31, 32). This delayed-onset-muscle-soreness (DOMS) is a common occurrence following bouts of unaccustomed strenuous physical activity and can result in muscle tenderness and debilitating pain symptoms (31). Thus, the training of new skills, and utilising often unused muscle groups, might have led to stressed muscles during training which was experienced by the participants as an increase in pain levels (32, 33). Several studies have reported on the relationship between the presence of pain and poor mood, reduced health and the ability of pain to interfere significantly with daily functioning (34, 35). This was also evident in the current study which showed an increase in the perception of pain interference with life domains over time, particularly for daily activities.

The shoulder has been reported to be the joint most commonly associated with pain above the level of injury in individuals with SCI (32, 36, 37). Due to the shoulder's complex functional anatomy and limited muscle mass, it is especially at risk for overuse injuries (32). Therefore, it is expected that the ABT group in our study rated the shoulder as the most painful area, due to the high levels of upper body utilization within this training modality. RLT participants may have also encountered upper body pain attributed to the performance of a new exercise modality that uses the upper extremities for support in the Ekso GT exoskeleton (38). However, in the current study, the RLT group rated the lower back as the site of greatest pain. Lower back pain is common within the SCI population with a prevalence of between 50% and 70% (36, 38) and may have been caused by the strain involved in maintaining upright posture and reaching the weights shifts required in the exoskeleton.

The second relevant finding of this pilot trial was that no changes in total spasticity were observed over the intervention period or between the groups. The beneficial effects that physical therapy modalities, such as ABT, have on spasticity in people with SCI is well-established (8, 17, 33, 39–42). Improvements in spasticity with the use of RLT have been speculated based on benefits previously described with body-weight-supported-treadmill training (BWSTT) (43). Regular standing and active exercises have both shown improvements in passive range of movement, posture, muscle strengthening and reduced stress and fatigue, which in turn aid in reducing spasticity symptoms (40). Both static and active standing may increase inhibition of the stretch reflex, reduce motor neuron excitability and subsequently reduce spasticity in individuals with SCI (40–42). Despite the proposed benefits of physical activity on spasticity, the current study showed no statistical changes in total spasticity scores between the RLT and ABT group or over time. However, there are no clear guidelines in the literature regarding the correct dosage and timing of exercise interventions required for these observed effects (22). Additionally, spasticity depends on the type, site, and duration of injury as well as other influencing factors that can trigger or aggravate spasticity symptoms, such as caffeine, lack of sleep, heat changes and pharmacological options (21, 22). These various contributing factors could be potential co-founding variables adding to the diverse spasticity responses found within this study.

The last notable finding in the current study is the increased perceptions of QoL for both the RLT and ABT group over time (Figure 4). These preliminary results add to previous findings that link engagement in physical activity with improved health and QoL (3). Standing and ambulation in particular, have been linked to improved psychological well-being and QoL in people with SCI (11, 44). Interestingly, despite the increasing levels of pain and minimal changes in spasticity reported in this study, QoL improved over time. Chronic pain is the most frequently reported reason for decreased QoL after SCI (45). However, this study indicates a dichotomous relationship between pain, spasticity and QoL among the participants. This may be due to the nature of the pain experienced by the participants, in that the pain was related to the demanding physical training and not as a complication of their SCI. Furthermore, although pain may have increased during the trial, the psychological benefits of partaking in the intervention, including standing and being active may have outweighed the effects of pain on QoL. There are many psychological and social benefits to standing, including improved self-image, eye-to-eye interpersonal contact and increased independence, all of which contribute to enhancing QoL (16). Wheelchair users may enjoy and value the normalizing experience of seeing themselves upright and participating in the walking motion (46). Thus, the increased QoL reported in this study could be attributed to the improved psychological benefit of standing and even walking again (47). For individuals with SCI, many ADLs are physical in nature (e.g., transferring, wheeling, eating). As a result, individuals who lack the physical capacity to perform basic ADLs may judge these tasks as stressful because of feelings of helplessness due to an inability to cope with the demands of daily living (35). Thus, the achievement of physical goals over the 24-week interventions may have also have led to increased perceptions of physical QoL, greater satisfaction with physical abilities and improved self-image and self-efficacy (48–50).

In addition, quality relationships and providing or receiving social and emotional support, can improve psychological well-being in people with SCI (48, 49, 51). Within the South African context, most individuals with SCI do not receive out-patient rehabilitation, let al.one the opportunity to exercise in a large multi-disciplinary training setting (52). Due to the lack of neurological specific rehabilitation centres in South Africa, most of the participants in this trial were unfamiliar with training in groups or alongside others with similar conditions. Consequently, the effect of regular social participation and interpersonal support, provided by the rehabilitation setting, may have led to improved psychological QoL for the participants in this study (53).

### 4.1. Limitations

As pain was a secondary analysis of the RCT, a limitation to the interpretation of the results is that analgesic/non-steroidal anti-inflammatory use was not documented. In addition, this study did not have a true experimental control in which no exercise was performed. Although an equivalent control group may be the experimental ideal, it is not feasible when conducting exercise interventions within the SCI population due to health and ethical implications. Although the small sample size was restrictive for statistical power and limits the generalisability of these findings to the larger SCI population, it may still provide important preliminary information for researchers to expand upon, as statistically insignificant changes could be of substantial clinical significance for people with SCI. An RCT with a larger sample size is warranted to further examine these findings.

### 4.2. Conclusion

In conclusion, it appears that neither intervention had an effect on reducing spasticity symptoms over 24-weeks. Pain ratings increased significantly for both interventions, though QoL perceptions were improved for both groups across the intervention period. This finding represents an interesting dichotomous relationship that requires additional investigation. Furthermore, the improved perceptions of QoL highlight the potential effectiveness of exercise interventions to support the well-being of people with SCI. Despite the small sample size, the adherence rate was extremely high and the cohort was homogenous, highlighting the strengths of this study and its contribution to the body of evidence on secondary health complications following SCI. A strong evidence base for the prevention and effective management of secondary complications will be essential for future breakthroughs in SCI health and well-being. Therefore, continued experimental research and rigorous studies are warranted to investigate these results further.

## Data Availability

The raw data supporting the conclusions of this article will be made available by the authors, without undue reservation.
