# Supplementary material for: Robotic locomotor training for spasticity, pain, and quality of life in individuals with chronic SCI: A pilot randomized controlled trial
Source: Front Rehabil Sci. 2023 Jan 30;4:1003360. doi: 10.3389/fresc.2023.1003360 (PMC9922844; doi:10.3389/fresc.2023.1003360)
Supplement: Supplementary file 1 [file Table1.docx]

**eTable 1: Inclusion and Exclusion Criteria**

Inclusion criteria

#### Male or female individuals between 18 and 65 years old

#### Chronic (>1yr) spinal cord injury

#### Motor incomplete (AIS C, D), with a neurological level of injury (NLI) between C1-C8 (tetraplegia) as determined by the International Standards for Neurological Classification of SCI (ISNCSCI)

- Reliant upon a wheelchair as the primary mode of mobility
- Sufficient anthropometrics and range of motion (ROM) to achieve a normal, reciprocal gait pattern within the Ekso GT^TM^ suit
- Medically stable and cleared by a physician for full weight bearing locomotor training including 15-minute standing frame trial to assess standing tolerance.

Exclusion criteria

- Non-traumatic SCI
- Have trained in a robotic exoskeleton in the past 12-months
- Performing any other form of locomotor training
- Psychopathology which may be exacerbated by inclusion into the trial
- Modified Ashworth Scale (MAS) = 4 in any of the lower extremity joints
- Skin integrity issues in areas that contact the device
- Pregnancy
- Z scores outside of recommended range (< -2)
- Any medical issue that in the opinion of the investigating team precludes full weight bearing locomotor training, including but not limited to:
  - Heart or respiratory comorbidity
  - Spinal instability (or spinal orthotic unless cleared by physician)
  - Acute deep vein thrombosis (DVT) with activity restrictions
  - Stoma bag
  - Severe, recurrent autonomic dysreflexia (AD) requiring medical intervention
  - Heterotopic ossification (HO) in the lower extremities resulting in ROM restrictions at the hips or knees
  - Two or more pathological fractures in the last 48 months in a major weight bearing bone (femur or tibia) in the lower extremity
  - Hip subluxation (x-rays will be obtained for individuals injured prior to 10 years of age.
- Any medical issue that in the opinion of the investigating team would affect participant safety either due to cognitive deficits/impulsivity, intolerance to mild exercise or other factors
- Any issue that in the opinion of the investigating team would confound results such as a concurrent neurological injury or disorder (other than SCI)
